# Exploring immune-inflammation markers in psoriasis prediction using advanced machine learning algorithms

**DOI:** 10.3389/fimmu.2025.1619490

**Published:** 2025-07-31

**Authors:** Li Yang, Shixin He, Li Tang, Xiao Qin, Yan Zheng

**Affiliations:** Department of Medical Cosmetology, The Third People’s Hospital of Chengdu, Chengdu, Sichuan, China

**Keywords:** psoriasis, national health and nutrition examination survey, machine learning algorithms, monocyte-to-lymphocyte ratio, neutrophil-to-monocyte ratio

## Abstract

**Background:**

Psoriasis is a chronic immune-mediated inflammatory skin disorder characterized by multifactorial pathogenesis. Recent studies have extensively highlighted the strong associations between psoriasis and various inflammatory markers, which are considered novel predictive tools for evaluating systemic inflammation.

**Methods:**

Cross-sectional data from the NHANES were analyzed in this study. To assess model performance and generalizability, the dataset was randomly divided into 70% for training and 30% for validation. To address class imbalance in the training data, a hybrid resampling technique (SMOTEENN) was applied. Subsequently, nine classification algorithms were developed using the processed training set, including random forest, neural networks, XGBoost, k-nearest neighbors, gradient boosting, logistic regression, naïve Bayes, AdaBoost, and SVMs. The final gradient boosting was implemented via the gbm package in R, with hyperparameters selected from the default tuning grid of the caret framework. Inflammatory biomarkers with the highest classification utility were identified based on the predictions of the best-performing model.

**Results:**

A total of 22,908 participants were included in the final analysis. Gradient boosting (AUC: 0.629, 95% CI: 0.588–0.669) demonstrated the highest performance, followed closely by logistic regression (AUC: 0.627, 95% CI: 0.588–0.666). Among all the inflammatory markers, MLR exhibited the best classification performance, with an AUC value of 0.662 (95% CI: 0.640–0.683), followed by NLMR, with an AUC value of 0.661 (95% CI: 0.640–0.683). Other markers, including the NLR, dNLR, SII, SIRI, and PLR, had AUC values ranging from 0.658 to 0.661. The MLR had the highest relative importance score, demonstrating its critical role in the model’s predictive performance for psoriasis classification. The NLR ranked second, followed by the SII and SIRI, which had moderate contributions, whereas the PLR contributed the least.

**Conclusions:**

Among all the tested algorithms, the gradient boosting model achieved the best performance. Not only does it achieve the highest predictive accuracy, but it also excels in classification efficacy and feature importance analysis, highlighting key inflammatory markers such as the MLR, SII, and NLR. These markers are significant as reliable indicators for evaluating systemic inflammation and predicting the development of psoriasis, emphasizing their potential clinical applications.

## Background

Psoriasis is a chronic skin disease, and studies have shown that its prevalence ranges from 0.51% to 11.43% in adults, whereas in children, it ranges from 0% to 1.37% (1). Regional statistics indicate that the lowest prevalence is observed in East Asia, at 0.14% (95% CI: 0.05%-0.40%), whereas Oceania has a relatively high prevalence of 1.99% (95% CI: 0.64%-6.60%). Additionally, high prevalence rates are observed in Western Europe (1.92%, 95% CI: 1.07%-3.46%), Central Europe (1.83%, 95% CI: 0.62%-5.32%), North America (1.50%, 95% CI: 0.63%-3.60%), and high-income regions of southern Latin America (1.10%, 95% CI: 0.36%-2.96%) (2). The incidence of psoriasis also exhibits a distinct bimodal age distribution, with the first peak occurring between 30 and 39 years of age and the second peak occurring between 60 and 69 years of age. Studies have suggested that females tend to develop psoriasis at an earlier age than males do. As age increases, the prevalence peaks between 60 and 70 years of age and subsequently decreases (3).

The pathogenesis of psoriasis involves genetic factors, immune system abnormalities, and various environmental triggers, such as infections, medication use, and psychological stress. In addition to cutaneous manifestations, the disease may also be associated with systemic inflammatory responses, including abnormalities in the cardiovascular, neurological, hepatic, and endocrine-metabolic systems (4, 5). Due to the systemic circulation of inflammatory cells and cytokines, studies have suggested that cutaneous inflammatory processes may contribute to dysfunction in systemic organs (6). The clinical manifestations of psoriasis are diverse, ranging from mild and stable forms to severe and active disease. Systemic involvement is common, accompanied by complications and significant elevations in parameters indicative of heightened innate immune activity, particularly in patients with severe active disease (7). Thus, analyzing inflammation-related markers is critical for assessing the dynamics of the disease and its systemic impact, providing valuable insights into disease severity and overall inflammatory status.

Recent studies have extensively highlighted the strong associations between psoriasis and various inflammatory markers, which are considered novel predictive tools for evaluating systemic inflammation. These markers include the Systemic Immune-Inflammation Index (SII) and the Systemic Inflammation Response Index (SIRI) (8–10). Paliogiannis et al., through a systematic review and meta-analysis, demonstrated that the NLR (neutrophil-to-lymphocyte ratio) and PLR (platelet-to-lymphocyte ratio) were significantly greater in psoriasis patients than in healthy controls (standard mean difference: SMD = 0.69, 95% CI: 0.53–1.85, p < 0.001; SMD = 0.40, 95% CI: 0.12–0.68, p = 0.006) (11). This finding was further validated by larger population studies, reinforcing the association between psoriasis and these inflammatory markers[ (12). In addition, other indices derived from complete blood cell counts have garnered attention, such as the dNLR (derived neutrophil-to-lymphocyte ratio), MLR (monocyte-to-lymphocyte ratio), NLMR (neutrophil-to-monocyte ratio), AISI (aggregate index of systemic inflammation), PWR (platelet-to-white blood cell ratio), and NPR (neutrophil-to-platelet ratio) (13–17). These markers are quick and straightforward to measure, allowing for repeated monitoring of disease progression or treatment effectiveness, making them particularly suitable for large-scale population screening and routine clinical monitoring.

In previous studies, multivariable linear regression and logistic regression models have been widely used to analyze the relationships between inflammatory markers and psoriasis (9, 18, 19). However, with the increasing complexity and dimensionality of data, these traditional statistical methods have gradually revealed their limitations, particularly in handling nonlinear relationships and interactions with high-dimensional data. In recent years, machine learning technologies (20, 21), with their superior data processing capabilities, have emerged as pivotal tools in medical research. Algorithms can effectively perform feature selection and optimization, uncover patterns hidden within complex datasets, and enhance the accuracy and efficiency of predictive models.

Therefore, the present study utilized cross-sectional data from the National Health and Nutrition Examination Survey (NHANES) to train multiple machine learning models, evaluate the predictive ability of various inflammatory markers, and ultimately identify optimal inflammatory biomarkers. To ensure the comprehensiveness of the research and the reliability of the results, nine machine learning algorithms were employed, including random forest, neural network, extreme gradient boosting (XGBoost), k-nearest neighbors, gradient boosting, logistic regression, naive Bayes, adaptive boosting (AdaBoost) and support vector machines (SVM). Finally, based on the predictions of the best-performing model, further evaluation was conducted to assess the predictive contributions of various inflammatory markers for psoriasis onset, leading to the identification of biomarkers with the highest clinical utility.

## Methods

### Study population

Our study utilizes data derived from the NHANES database, which serves as a comprehensive, ongoing nationwide health and nutrition monitoring program. The NHANES database originated in the 1960s and, since 1999, has been used in biennial cycles to ensure the randomness and representativeness of the samples, thereby accurately reflecting the health status and behavioral characteristics of the U.S. population (official website link: https://wwwn.cdc.gov/nchs/nhanes/Default.aspx). The NHANES dataset encompasses an extensive array of multidisciplinary information, including demographic details, dietary intake and deficiencies, biological concentrations of nutritional biomarkers, health behaviors, and key indicators obtained through laboratory tests and physical examinations. Prior to data collection, all participants provided written informed consent, and Institutional Review Board approval was obtained. For this study, we specifically focused on NHANES data related to psoriasis, which were primarily concentrated within the periods of 2003–2006 and 2009–2014, and incorporated these datasets into our in-depth analysis.

**Figure 1:**
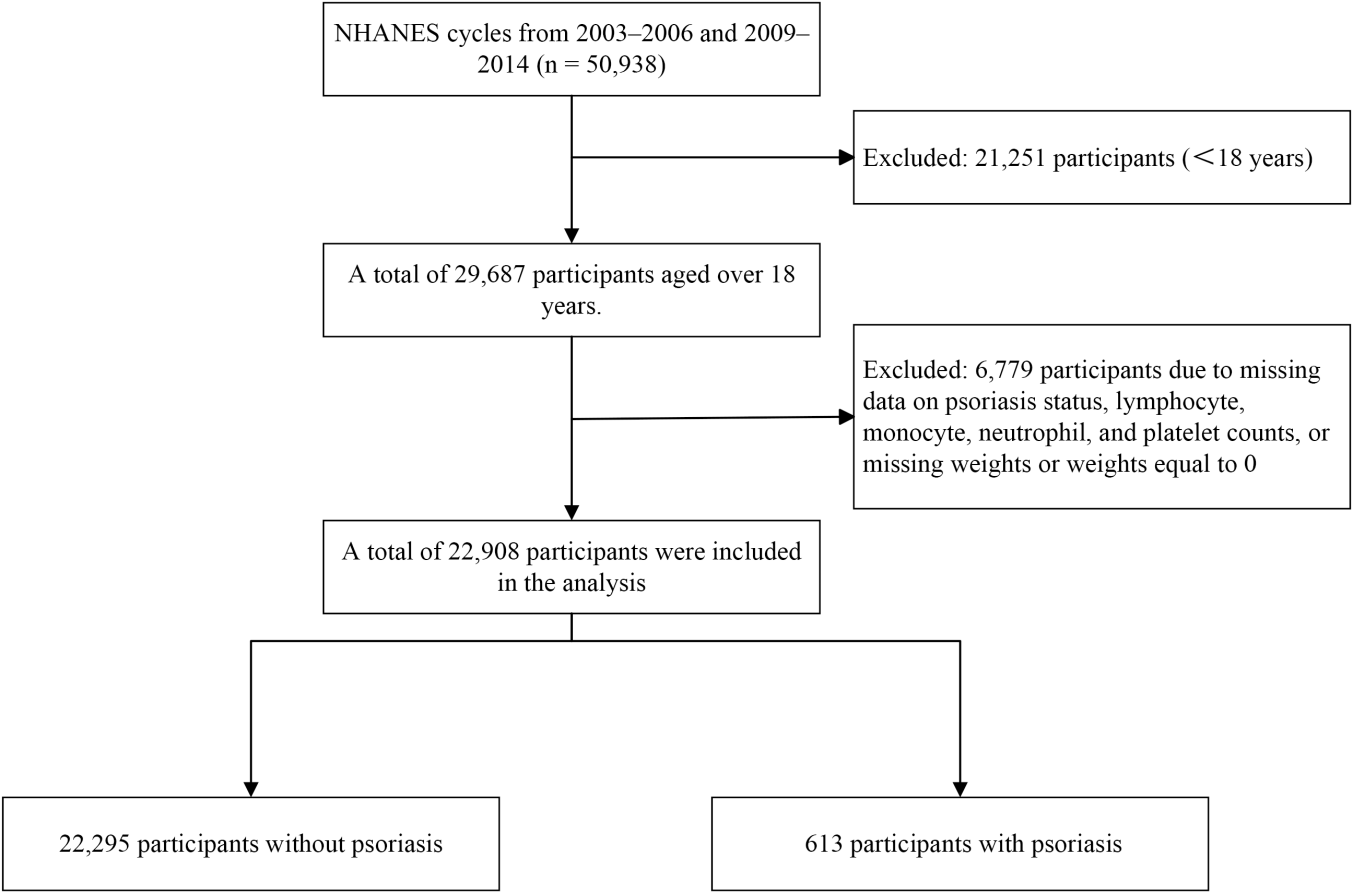
Flowchart illustrating the participant selection process in NHANES cycles.

The 2003–2006 NHANES cycles included individual interview data that provided information on psoriasis, sun exposure, and sunscreen usage. The participants were asked the following question: “Have you ever been told by a health care provider that you had psoriasis?” Similarly, during the 2009–2014 NHANES cycles, self-reported data on various health conditions were collected, using the same question to identify patients with psoriasis. Across these cycles, a total of 50,938 participants were included (**Figure 1**). The exclusion criteria for this sample were as follows: participants under 18 years of age (n = 21,251) and participants with missing data, including psoriasis status, lymphocyte count, monocyte count, neutrophil count, platelet count, or missing weights/weights equal to 0 (n = 6,779); a total of 22,908 participants were retained for analysis.

### Index calculation and data collection

The following hematological and inflammatory indices were calculated via detailed methods based on cellular counts and ratios derived from laboratory measurements:

1. NLR: The NLR represents the ratio of neutrophils to lymphocytes and is calculated as follows: NLR = neutrophil count ÷ lymphocyte count. This index is widely utilized to assess systemic inflammation, as neutrophils are key players in acute immune responses, whereas lymphocytes represent adaptive immunity.
2. dNLR: The dNLR provides an alternative to the NLR, with a focus on derived white blood cell subsets. It is calculated as follows: dNLR = neutrophil count ÷ (total white blood cell count − neutrophil count). This adjustment accounts for the exclusion of neutrophils from the total white blood cell population, emphasizing the derived inflammatory profile.
3. SII: The SII combines neutrophils, lymphocytes, and platelets to reflect systemic immune and inflammatory status. It is calculated as follows: SII = (platelet count × neutrophil count) ÷ lymphocyte count. This index is particularly relevant for evaluating inflammation related to immune responses and disease progression.
4. SIRI: The SIRI incorporates monocytes along with neutrophils and lymphocytes, representing a broader measure of inflammation. It is calculated as follows: SIRI = (neutrophil count × monocyte count) ÷ lymphocyte count. This index provides insights into the inflammatory responses driven by innate immune cells.
5. MLR: The MLR reflects the balance between monocytes and lymphocytes and is calculated as follows: MLR = monocyte count ÷ lymphocyte count. Monocytes play a significant role in chronic inflammation, making this ratio valuable for understanding immune dynamics.
6. PLR: The PLR reflects the relationship between platelets and lymphocytes and is calculated as follows: PLR = platelet count ÷ lymphocyte count. Platelets contribute to both hemostasis and inflammatory processes, whereas lymphocytes represent adaptive immunity.
7. NLMR: The NLMR integrates neutrophils and monocytes into a single ratio relative to lymphocytes and is calculated as NLMR = (neutrophil count + monocyte count) ÷ lymphocyte count. This index captures the combined effects of innate immune cells in the context of systemic inflammation.
8. AISI: The AISI reflects a comprehensive measure of systemic inflammation by incorporating neutrophils, monocytes, and platelets, calculated as follows: AISI = (Neutrophil count × Monocyte count × Platelet count) ÷ Lymphocyte count. This aggregate index offers a multidimensional view of inflammatory activity.
9. PWR: The PWR focuses on the balance between the PLT and total white blood cell count, which is calculated as follows: PWR = platelet count ÷ total white blood cell count. This ratio highlights platelet involvement relative to total leukocytes in immune responses.
10. NPR: The NPR reflects the relationship between neutrophils and platelets and is calculated as follows: NPR = neutrophil count ÷ platelet count. This index sheds light on the interplay between inflammatory cells and thrombocytes.

A wide range of variables across demographics, lifestyle factors, comorbidities, and health metrics exist. The demographic data included age, race, educational status, marital status, and the family poverty income ratio (PIR). Lifestyle factors include drinking habits and smoking history, whereas comorbidities include hypertension, diabetes, cardiovascular disease (CVD), and osteoarthritis (OA). Health metrics include systolic blood pressure (SBP), diastolic blood pressure (DBP), body mass index (BMI), fasting plasma glucose (FPG), HbA1c, total cholesterol (TC), high-density lipoprotein cholesterol (HDL-C), triglycerides (TG), and low-density lipoprotein cholesterol (LDL-C). Additionally, blood metrics such as platelet count, segmented neutrophils, lymphocytes, monocytes, and white blood cell counts are presented. Furthermore, variables with more than 15% missing data were excluded to ensure the reliability of the analysis (**Supplementary Table S1**).

### Statistical analysis

First, variables with less than 15% missing values were included in the final analysis. Missing data were efficiently handled via the `mice` package in R through the multiple imputation by chained equations (MICE) method. Appropriate regression models, such as linear regression for continuous data and logistic regression for categorical data, were applied according to the variable types. The iterative sampling and imputation processes were performed via the Markov chain Monte Carlo (MCMC) algorithm. Variables were processed sequentially using the fully conditional specification (FCS) approach, which leveraged both existing values and imputed values to estimate missing data, thereby ensuring both flexibility and accuracy in the imputation process.

The analysis utilized functions to construct complex survey design objects by specifying stratification variables, cluster variables, weight variables, and adjustment options for complex survey sampling. The continuous variables in **Table 1** are presented as means and standard deviations and were analyzed using t-tests, whereas the categorical variables are presented as frequencies and percentages and were analyzed using chi-square tests. The evaluation of linear relationships was conducted by calculating the Pearson correlation coefficients between variables. An absolute correlation coefficient close to 1 indicates the potential presence of a significant linear correlation among variables. However, relying solely on correlation coefficients is insufficient to detect issues of multicollinearity fully. Therefore, this study further employed the variance inflation factor (VIF) for detailed analysis. The VIF values of each predictor variable were calculated individually to determine whether multicollinearity existed between it and other variables. Generally, a VIF value exceeding five is considered indicative of significant multicollinearity, suggesting that model adjustments may be necessary to enhance robustness.

**Table 1:** Baseline demographic and clinical characteristics of participants.

Variable	Overall, N = 22908 (100%)^1^	No psoriasis, N = 22295 (97%)^1^	With psoriasis, N = 613 (3.0%)1	*P*-value
Age, years	44.18(16.05)	44.08(16.07)	47.39(15.08)	<0.001
Sex, N (%)				0.7
female	11,026 (49%)	10,739 (49%)	287 (48%)	
male	11,882 (51%)	11,556 (51%)	326 (52%)	
Race, N (%)				<0.001
Mexican American	3,724 (8.8%)	3,673 (9.0%)	51 (3.8%)	
Other Hispanic	1,889 (5.2%)	1,841 (5.2%)	48 (4.0%)	
Non-Hispanic White	10,078 (68%)	9,703 (67%)	375 (81%)	
Non-Hispanic Black	4,904 (11%)	4,828 (11%)	76 (5.8%)	
Other/multiracial	2,313 (6.9%)	2,250 (7.0%)	63 (5.6%)	
Educational status, N (%)				0.3
Less Than 9th Grade	2,219 (5.3%)	2,181 (5.4%)	38 (3.0%)	
9-11th Grade	3,412 (11%)	3,328 (11%)	84 (11%)	
High School Grad/GED	5,121 (22%)	4,984 (23%)	137 (21%)	
Some College or AA degree	6,961 (32%)	6,763 (32%)	198 (34%)	
College Graduate or above	5,163 (28%)	5,007 (28%)	156 (30%)	
Refused answer	17 (<0.1%)	17 (<0.1%)	0 (0%)	
Don't Know	15 (<0.1%)	15 (<0.1%)	0 (0%)	
Marital Status, N (%)				0.8
Married	11,529 (55%)	11,217 (55%)	312 (57%)	
Widowed	1,386 (4.3%)	1,344 (4.3%)	42 (5.0%)	
Divorced	2,303 (10%)	2,222 (10%)	81 (11%)	
Separated	752 (2.4%)	728 (2.4%)	24 (2.7%)	
Never married	4,950 (20%)	4,848 (20%)	102 (17%)	
Living with partner	1,967 (8.2%)	1,915 (8.2%)	52 (7.8%)	
Refused answer	11 (<0.1%)	11 (<0.1%)	0 (0%)	
Don't Know	10 (<0.1%)	10 (<0.1%)	0 (0%)	
Family PIR	2.95(1.66)	2.95(1.66)	3.08(1.67)	0.087
Drinking habit, N (%)				0.007
1-5 drinks/month	6,528 (23%)	6,376 (23%)	152 (20%)	
10+ drinks/month	11,412 (51%)	11,110 (51%)	302 (46%)	
5-10 drinks/month	1,850 (9.5%)	1,789 (9.4%)	61 (13%)	
Non-drinker	3,118 (17%)	3,020 (17%)	98 (21%)	
Smoking history, N (%)				<0.001
Current smoker	12,847 (55%)	12,575 (55%)	272 (43%)	
Former smoker	4,937 (22%)	4,740 (22%)	197 (35%)	
Never smoker	5,124 (23%)	4,980 (23%)	144 (21%)	
Hypertensions, N (%)				<0.001
Yes	14,416 (66%)	14,099 (66%)	317 (55%)	
No	8,492 (34%)	8,196 (34%)	296 (45%)	
SBP, mmHg	120.54(16.45)	120.50(16.45)	121.57(16.39)	0.063
DBP, mmHg	70.48(12.06)	70.44(12.06)	71.86(11.84)	0.013
BMI (kg/m2)	98.07(16.47)	97.95(16.45)	101.97(16.74)	<0.001
HbA1c, %	5.54(0.89)	5.54(0.90)	5.57(0.81)	0.021
TC, mmol/L	5.03(1.07)	5.03(1.07)	5.12(1.07)	0.14
HDL-C, mmol/L	1.38(0.41)	1.38(0.41)	1.34(0.41)	0.035
Platelet count (1000 cells/uL)	250.3815(65.7540)	250.3104(65.6891)	252.6618(67.8198)	0.4
Segmented neutrophils num (1000 cell/uL)	4.3400(1.7484)	4.3345(1.7503)	4.5176(1.6775)	0.017
Lymphocyte number (1000 cells/uL)	2.1269(0.8714)	2.1296(0.8768)	2.0399(0.6720)	0.025
Monocyte number (1000 cells/uL)	0.5501(0.1965)	0.5500(0.1974)	0.5562(0.1652)	0.15
White blood cell count (1000 cells/uL)	7.2624(2.2383)	7.2588(2.2438)	7.3753(2.0558)	0.2
NLR	2.22(1.14)	2.21(1.14)	2.42(1.28)	<0.001
dNLR	1.56(0.69)	1.55(0.68)	1.66(0.75)	<0.001
SII	555.78(342.97)	554.05(342.51)	611.20(353.27)	<0.001
SIRI	1.24(0.87)	1.24(0.88)	1.36(0.83)	<0.001
MLR	0.28(0.12)	0.28(0.12)	0.30(0.12)	<0.001
PLR	128.21(48.78)	128.00(48.65)	134.97(52.54)	0.01
NLMR	2.49(1.21)	2.49(1.21)	2.72(1.35)	<0.001
AISI	314.82(262.85)	313.84(263.51)	346.16(238.80)	<0.001
PWR	36.58(11.95)	36.60(11.98)	35.98(11.08)	0.6
NPR	0.0182(0.0105)	0.0181(0.0106)	0.0188(0.0082)	0.066

^1^Mean (SD); n (unweighted) (%).

PIR, Poverty-to-Income Ratio; BMI, Body Mass Index; SBP, Systolic Blood Pressure; DBP, Diastolic Blood Pressure; HbA1c, Glycated Hemoglobin; TC, Total Cholesterol; HDL-C, High-Density Lipoprotein Cholesterol; NLR, Neutrophil-to-Lymphocyte Ratio; dNLR, Derived Neutrophil-to-Lymphocyte Ratio; SII, Systemic Immune-Inflammation Index; SIRI, Systemic Inflammation Response Index; MLR, Monocyte-to-Lymphocyte Ratio; PLR, Platelet-to-Lymphocyte Ratio; NLMR, Neutrophil-to-Lymphocyte and Monocyte-to-Lymphocyte Ratio; AISI, Aggregate Index of Systemic Inflammation; PWR, Platelet-to-White Blood Cell Ratio; NPR, Neutrophil-to-Platelet Ratio.

For further model analysis, stratified random sampling was used to divide the dataset into 70% training data and 30% validation data. To address class imbalance, SMOTEENN random oversampling techniques were applied to balance the training dataset. This method generates additional samples based on the distribution of the original data, notably by expanding instances of minority classes to mitigate imbalance. Subsequently, noise reduction was performed via the k-nearest neighbors (k-NN) algorithm. For each sample, the k-NN algorithm computes the distance between the sample and its nearest neighbors and applies majority voting to predict the class of the sample. This step eliminates potential noise samples with incorrect classifications, retaining only those that are correctly classified to optimize data quality.

After the data were balanced, multiple machine learning models, including random forest, neural network, XGBoost, k-nearest neighbors, gradient boosting, logistic regression, Naibe Bayes, AdaBoost, and SVM, were trained. Predictive probabilities were generated on the test set, and model performance in distinguishing positive and negative samples was evaluated via receiver operating characteristic (ROC) curves. ROC curves for different models were plotted, and the area under the curve (AUC) values for each model were calculated as key indicators of model performance. The closer the AUC is to 1, the stronger the model’s classification ability. Additionally, accuracy, precision, recall, and F1-scores were computed for each model to highlight differences in classification ability and performance across the machine learning approaches. To further evaluate model fit, the Hosmer–Lemeshow goodness-of-fit test was employed. The results revealed that P > 0.05 indicates a good fit, suggesting strong consistency between the model predictions and observed values, whereas *P* ≤ 0.05 suggests significant discrepancies, potentially indicating poor fit.

Finally, based on the complex sampling design, the feature importance of inflammatory markers from the selected optimal model was ranked, and AUC values were calculated using ROC curves to quantify the classification effectiveness of each metric. To evaluate the subgroup-specific relevance of inflammatory markers, SHapley Additive exPlanations (SHAP) analysis was performed on the optimal model. Feature contributions were visualized with color-coding based on four sociodemographic variables: age group (≤60 years vs. >60 years), sex (Male vs. Female), race/ethnicity (Mexican American, Other Hispanic, Non-Hispanic Black, Other Race, Non-Hispanic White), and income level (Below Poverty [PIR ≤ 1], Near Poverty [1 < PIR ≤ 2], Middle Income [2 < PIR ≤ 4], High Income [PIR > 4]). All the analyses were conducted in R version 4.4.2.

## Results

A total of 22,908 participants were included in the final analysis, with an average age of 44.18 years. Patients with psoriasis were significantly older, with a mean age of 47.39 years, and this difference was statistically significant (*P* < 0.001). No significant differences were observed between the groups in terms of sex distribution (*P* = 0.7) or marital status (*P* = 0.8). Compared with nonpsoriatic patients, individuals with psoriasis accounted for a greater proportion of nondrinkers and a significantly greater proportion of former smokers. Among other health indicators, patients with psoriasis presented higher DBP and HbA1c levels than the controls, with *P* values of 0.013 and 0.021, respectively. While TC levels were not significantly different (*P* = 0.14), high HDL-C levels were significantly lower in patients with psoriasis (*P* = 0.035). Furthermore, inflammation and immune-related indices, including the NLR, dNLR, SII, SIRI, and MLR, were markedly elevated in patients with psoriasis (P < 0.001), suggesting that these indices may be strongly correlated with the presence of psoriasis. Additionally, as the PWR and NPR did not differ significantly between the psoriasis and non-psoriasis groups in the baseline table, these two metrics were also excluded during model construction to optimize performance.

**Figure 2:**
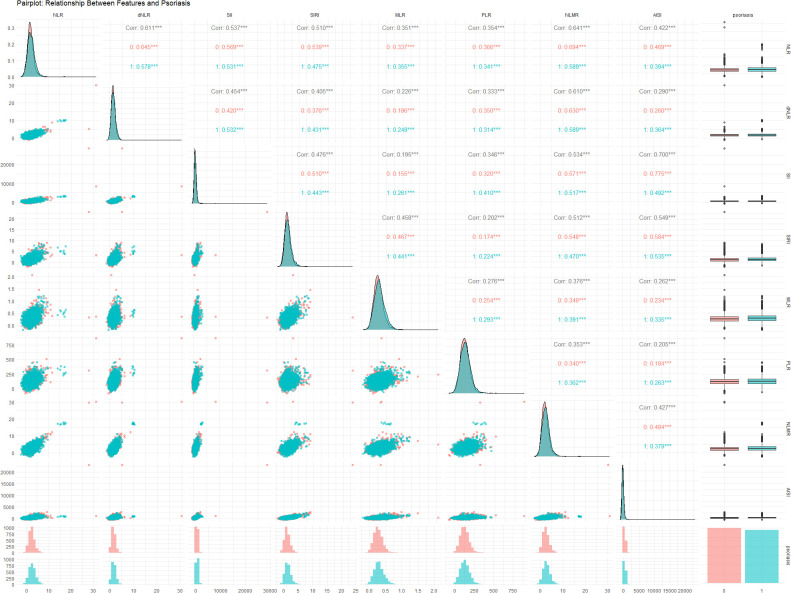
Relationships between the NLR, dNLR, SII, SIRI, MLR, PLR, NLMR, and AISI and psoriasis status. Each variable’s contribution and interaction within the data are analyzed to provide insights into their predictive capabilities and potential correlations. Symbols *, **, and *** indicate statistical significance levels: * p < 0.05, ** p < 0.01, *** p < 0.001.

**Figure 3:**
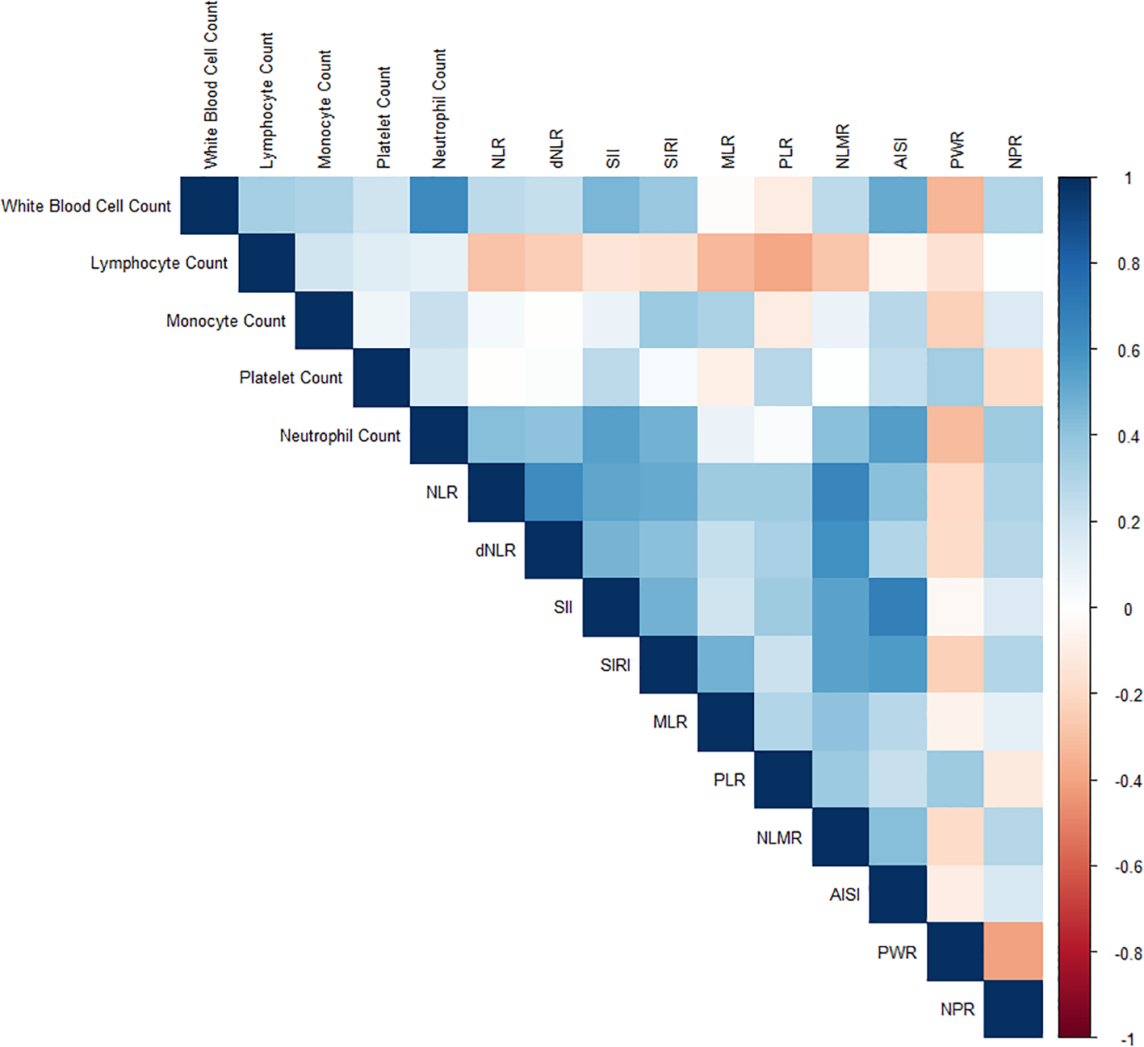
Correlation coefficient plot illustrating the relationships among various inflammatory markers. The coefficients are represented by a color gradient, with darker hues indicating stronger correlations.

**Figure 2** illustrates the relationships between various inflammatory markers and psoriasis. These inflammatory markers were derived from a fundamental whole-blood cell analysis. To further clarify the correlations among variables, a collinearity analysis was performed. The variance inflation factors (VIFs) of these inflammatory markers were all less than 5 (**Supplementary Table S2**), indicating relatively low multicollinearity among the variables. However, some markers, such as the SII (2.692) and AISI (2.526), have relatively high variance inflation factor (VIF) values, suggesting a certain degree of correlation with other variables. The correlation coefficient plot (**Figure 3**) reveals that although all the coefficients are less than 0.8, the dNLR and NLR, the SII and AISI, and the NLMR and NLR are highly correlated. Therefore, in the subsequent model training processes, dNLR, AISI, and NLMR were excluded from the analysis.

### Assessment of model performance

**Figure 4:**
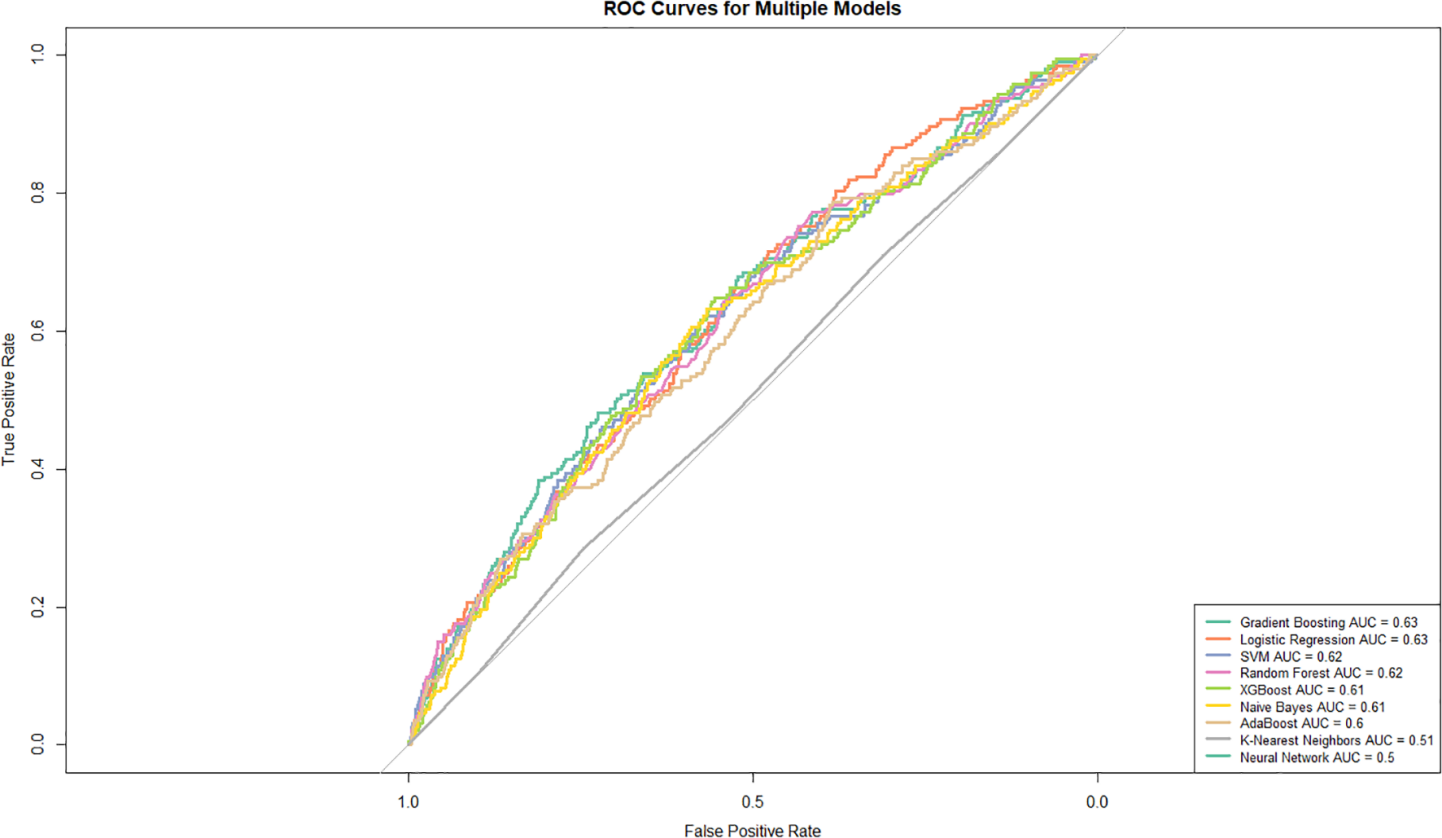
Receiver operating characteristic (ROC) curves for various models. The true positive rate (TPR) is plotted against the false positive rate (FPR) for each model. The curves highlight the area under the curve (AUC) values, providing a comparative measure of model performance.

**Figure 4** presents the rankings of the predictive models in descending order based on their AUC values. Gradient boosting (AUC: 0.629, 95% CI: 0.588–0.669) demonstrated the highest performance, followed closely by logistic regression (AUC: 0.627, 95% CI: 0.588–0.666). SVM (AUC: 0.618, 95% CI: 0.577-0.660) and random forest (AUC: 0.617, 95% CI: 0.576-0.658) algorithms also demonstrated competitive performance. XGBoost (AUC: 0.615, 95% CI: 0.574–0.655) and naive Bayes (AUC: 0.608, 95% CI: 0.567–0.649) showed moderate performance, followed by AdaBoost (AUC: 0.601, 95% CI: 0.560–0.643). K-nearest neighbors (AUC: 0.512, 95% CI: 0.471 - 0.553) and the neural network (AUC: 0.500, 95% CI: 0.500 - 0.500) displayed the lowest predictive ability in this analysis.

**Supplementary Table S3** presents the performance metrics of various machine learning models for classification analysis. The neural network demonstrated the best performance, achieving an accuracy of 0.972, a recall of 1, and the highest overall F1 score of 0.986. XGBoost and random forest closely followed, with commendable F1 scores of 0.883 and 0.871, respectively. AdaBoost also exhibited stable performance, with an F1 score of 0.87. Gradient boosting and SVM showed slightly weaker performance, achieving accuracies of 0.748 and 0.741, respectively, while maintaining consistently high precision. In contrast, logistic regression and naive Bayes demonstrated relatively average accuracies and F1 scores of 0.67 and 0.718, respectively. Finally, K-nearest neighbors underperformed, with the lowest accuracy of 0.534, as well as the lowest recall and F1 score, at 0.536 and 0.691, respectively. Furthermore, the Hosmer–Lemeshow chi-square test (**Supplementary Table S4**) revealed that only gradient boosting and logistic regression had *P* values greater than 0.05. Gradient boosting yielded a test statistic of 3.023 with a p-value of 0.08, whereas logistic regression produced a test statistic of 1.091 with a p-value of 0.3. Based on a comprehensive assessment, gradient boosting was selected as the optimal model.

### Classification performance of inflammatory markers

**Figure 5:**
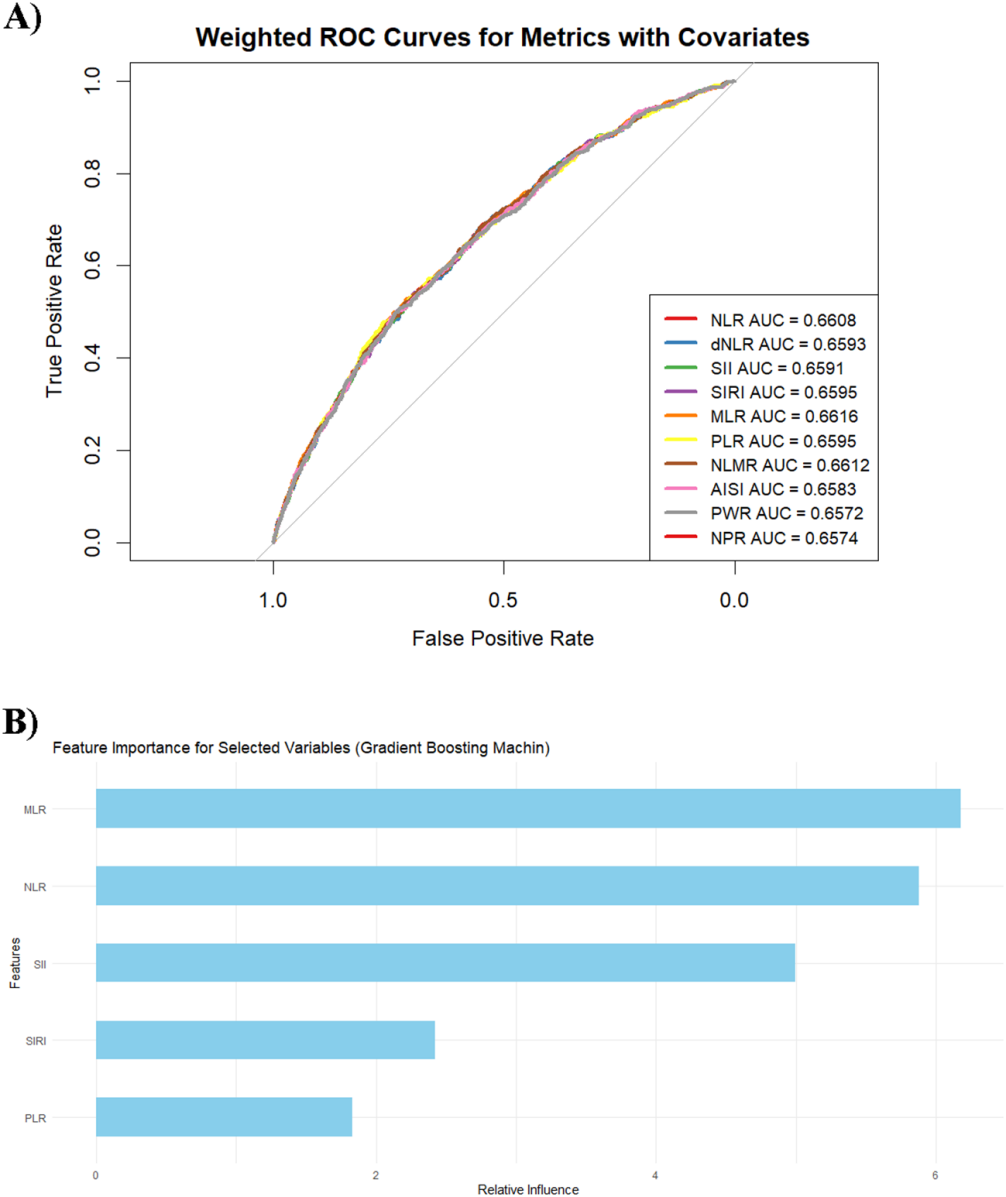
**(A)** Weighted ROC curves for the metrics with covariates. Each curve represents the classification effectiveness of the corresponding metric for predicting the target variable, adjusted for covariates such as age, sex, ethnicity, marital status, the family income–to–poverty ratio (PIR), BMI, waist circumference, glucose levels, lipid levels, alcohol consumption, smoking status, and hypertension. **(B)** Feature importance of the variables in the gradient boosting machine model.

Among all the inflammatory markers, the MLR exhibited the best classification performance, with an AUC value of 0.662 (95% CI: 0.640–0.683), highlighting its excellent efficacy in predicting psoriasis occurrence (**Figure 5A**). The NLMR has an AUC value of 0.661 (95% CI: 0.640–0.683), indicating outstanding performance. Other markers, including the NLR, dNLR, SII, SIRI, and PLR, had AUC values ranging from 0.658 to 0.661, indicating relatively consistent classification performance. In contrast, AISI, PWR, and NPR have lower AUC values, ranging from 0.657 to 0.658, suggesting weaker classification effectiveness. To further evaluate the predictive contribution of inflammatory markers, this study employed a gradient boosting machine learning model to analyze the feature importance of five core variables (MLR, NLR, SII, SIRI, and PLR) (**Figure 5B**). The analysis revealed that the MLR had the highest relative importance score, demonstrating its critical role in the model’s ability to predict psoriasis. The NLR ranked second, followed by the SII and SIRI, which had moderate contributions, whereas the PLR contributed the least. Moreover, SHAP dependence (**Supplementary Figure S1**) revealed consistent predictive effects of inflammatory markers across age, sex, race/ethnicity, and income levels. The absence of substantial SHAP value divergence across groups suggests that baseline demographic factors did not materially alter marker behavior within the model.

## Discussion

This study applied machine learning techniques to explore predictive models for psoriasis and revealed that the gradient boosting model demonstrated superior performance among all the models. In the model analysis, the inflammatory marker MLR had the highest feature importance score and the highest AUC value among all the markers in terms of classification performance. These findings underscore the pivotal role of the MLR in predicting psoriasis and validate its potential as a key marker for assessing systemic inflammation.

This study leveraged a large dataset and employed machine learning techniques to develop predictive models for psoriasis. By addressing the limitations of traditional statistical methods in handling variable interactions and nonlinear data, machine learning has demonstrated superior capabilities. Unlike conventional approaches, machine learning excels in capturing complex intervariable relationships, significantly enhancing predictive performance and model stability. Among the algorithms tested, the gradient boosting model proved to be the most effective, outperforming the other methods in terms of predictive accuracy. This study also highlighted the importance of key inflammatory markers, such as the tMLR, in feature importance analysis and classification efficacy. Previous studies have extensively investigated the application of machine learning in psoriasis research. For example, machine learning has been utilized to analyze biological markers of psoriasis, including inflammatory parameters, immune-related factors, and metabolic molecules, shedding light on the underlying disease mechanisms. Additionally, other studies have focused on pathway analysis and the identification of potential key genes, aiming to elucidate the genetic and molecular biology of psoriasis (22–24). As technology has advanced, the scope of machine learning applications in psoriasis research has broadened considerably. For example, one study summarized the progress spanning areas from skin image analysis to clinical management (25), including automated lesion identification and differential diagnosis, precise lesion segmentation, and severity and area scoring. However, these investigations have focused predominantly on image-based analyses or the exploration of disease mechanisms. In contrast, this study integrates classical machine learning algorithms with inflammatory markers to establish predictive models for psoriasis with novel clinical utility. To further evaluate the predictive role of these markers, nine established machine learning algorithms were selected, comprising both linear methods (e.g., logistic regression) and nonlinear approaches (e.g., gradient boosting and random forest). These models are capable of processing various data types and accounting for variable interactions, enabling systematic evaluations that optimize prediction performance. In particular, the MLR, an emerging inflammatory marker, demonstrated exceptional performance in this study.

The MLR is an emerging inflammatory marker that reflects the dynamic balance between monocytes and lymphocytes in peripheral blood, thereby serving as an indicator of the body’s inflammatory state. Research has established a significant association between the MLR and psoriasis. For example, a meta-analysis conducted by Liu et al. revealed that MLR values were significantly higher in psoriasis patients than in healthy controls (MD = 0.034, 95% CI: 0.021–0.048, P < 0.001) (14). Hagino et al. reported that the novel antibody Bimekizumab, which targets IL-17A and IL-17F, was associated with lower MLR values in long-term responders than in nonresponders, with long-term responders also tending to be younger (26). Additionally, the MLR has been associated with disease severity in patients with psoriasis. A retrospective study demonstrated a positive correlation between MLR parameters and the Psoriasis Area and Severity Index (PASI) (r = 0.153, *P* < 0.001) (27). Another study identified the MLR as an independent inflammatory marker for predicting the severity of liver fibrosis, a comorbidity of psoriasis (P < 0.001) (17). Furthermore, prior research has associated elevated MLR values with all-cause mortality in patients with psoriasis (13).

In addition to the established clinical associations of MLR, a mechanistic understanding of MLR may further substantiate its utility as a biomarker in psoriatic inflammation. Monocytes and M1-polarized macrophages are recognized as key producers of interleukin-23 (IL-23), a cytokine critical for the differentiation and activation of Th17 cells. The IL-23–Th17 axis (28, 29) plays a central role in the pathogenesis of psoriasis by promoting IL-17 secretion and keratinocyte-driven inflammation. Although MLR is a peripheral blood marker that does not directly reflect macrophage polarization, an elevated MLR may suggest increased monocyte-driven inflammation. Given that M1 macrophages are a major source of IL-23 in psoriatic lesions (30), it is plausible that higher MLR values could be associated with enhanced IL-23–Th17 axis activity. For example, a study demonstrated that a lower baseline MLR was associated with a better long-term response to bimekizumab, a dual IL-17A/F inhibitor, suggesting that MLR may reflect the degree of upstream monocyte-driven inflammation and Th17 axis activation (26). Furthermore, Demirel Öğüt et al. (31) reported that IL-17 inhibitors significantly reduced MLR and other systemic inflammatory markers, whereas IL-23 inhibitors did not, implying that MLR may be more closely linked to IL-17–mediated inflammation. These findings collectively support the notion that MLR may serve as a peripheral surrogate for monocyte-derived IL-23–Th17 axis activity in psoriasis.

Notably, psoriasis is a clinically heterogeneous condition, encompassing diverse subtypes and patient profiles that may influence the inflammatory signature and the relevance of biomarkers. Factors such as age, sex, lifestyle, and comorbidities can modulate both immune responses and disease manifestation. As such, the associations observed between inflammatory markers and psoriasis in this study may vary across distinct patient subgroups. Future research could benefit from exploring heterogeneity through subphenotyping or unsupervised clustering approaches, as demonstrated in the work of Yang et al. (32) in the context of sepsis. Such stratified analyses may offer deeper insights into patient-specific patterns and help refine individualized prediction models.

In contrast, other inflammatory indices, such as the SII and the NLR, have also been extensively studied in the context of psoriasis development and progression. Studies by Ma et al. (33), Zhao et al. (18), and Yorulmaz et al. (34) reported that the SII is a favorable indicator of the inflammatory status and immune response in psoriasis patients. Additionally, the SII has been observed to display nonlinear associations with psoriasis in specific subgroups, such as participants aged 20–39, former smokers, current alcohol users, individuals with or without a history of myocardial infarction, those without coronary artery disease, and overweight participants (35). Beyond its role as a general inflammatory marker, elevated SII may also reflect platelet-driven inflammation and endothelial activation in psoriasis. Platelets are increasingly recognized as active mediators in psoriatic inflammation, particularly in severe subtypes such as erythrodermic and pustular psoriasis. Activated platelets release proinflammatory cytokines, including interleukin-1β (IL-1β) and C-X-C motif chemokine ligand 8 (CXCL8, also known as interleukin-8 or IL-8), and interact with endothelial cells to promote vascular inflammation. Garshick et al. (36) demonstrated that platelets from psoriasis patients induce endothelial inflammation via cyclooxygenase-1 (COX-1)- dependent pathways, upregulating IL-1β, IL-8, and COX-2 expression in human aortic endothelial cells, with disease severity correlating with platelet activation levels. Visser et al. (37) further confirmed that psoriatic disease is associated with endothelial dysfunction and a prothrombotic state, characterized by elevated soluble adhesion molecules and dense fibrin networks. Some population-based studies have supported this hypothesis, showing that SII is independently associated with psoriasis risk (18, 19, 35) and correlates with comorbidities such as metabolic syndrome and all-cause mortality (10). These findings support the notion that SII may capture platelet-driven inflammatory and endothelial processes in psoriasis, beyond its role as a general inflammatory index.

Moreover, the NLR has demonstrated significant predictive value. For example, Kommoss et al. validated the NLR as an objective biomarker of skin inflammation in both psoriasis patients and preclinical models of psoriasis (38). These findings underscore the potential clinical applications of these markers. Psoriatic lesions are characterized by intense neutrophil infiltration and activation, including an oxidative burst and the formation of neutrophil extracellular traps (NETs) (39), which contribute to IL-17 release and keratinocyte activation (39, 40). An elevated NLR may therefore reflect not only increased neutrophil counts but also their heightened activation status. This is supported by recent studies demonstrating that NLR correlates with disease severity and systemic inflammatory burden in patients with psoriasis. For instance, Çelik et al. (41) reported that both IL-17 and IL-23 inhibitors significantly reduced NLR and SII after 6 months of treatment, suggesting that NLR is sensitive to therapeutic modulation of neutrophil-driven inflammation. Similarly, Kearney et al. showed that guselkumab treatment led to significant reductions in NLR, PLR, and MLR across three randomized clinical trials (42). These findings align with the notion that NLR not only quantifies neutrophil burden but may also indirectly capture functional activation states related to psoriatic inflammation. Moreover, accumulating evidence suggests that elevated NLR and SII values may reflect not only systemic inflammation but also the activity of the IL-23/IL-17 axis, which is central to the immunopathogenesis of psoriasis. IL-23 promotes the differentiation and maintenance of Th17 cells, which secrete IL-17A, a cytokine that drives neutrophil recruitment, keratinocyte activation, and chronic inflammation. IL-17A is produced not only by Th17 cells but also by neutrophils and mast cells in psoriatic lesions, forming a feed-forward loop that amplifies neutrophilic inflammation (43, 44). In this context, elevated NLR and SII may serve as peripheral surrogates of Th17 axis activity, capturing both the quantitative and functional aspects of neutrophil-driven inflammation. As previously noted (41), NLR and SII levels decrease in response to IL-17/IL-23–targeted therapies, further supporting their relevance to this cytokine axis. In addition, baseline NLR and SII values have been shown to positively correlate with PASI scores—a clinical index known to reflect IL-17/IL-23 activity (27). These findings suggest that NLR and SII are not merely general inflammatory markers but may indirectly capture the functional status of the IL-23/Th17 axis.

The MLR, SII, and NLR are all derived from routine blood test data and serve as crucial inflammatory markers with significant clinical utility in evaluating immune function and systemic inflammation. Specifically, the MLR reflects monocyte proliferation and activity while assessing lymphocyte-mediated immune regulation, providing insights into the body’s inflammatory balance. SII, which integrates platelet, neutrophil, and lymphocyte counts, offers a comprehensive evaluation of systemic inflammation and tumor-related immune responses. The NLR primarily reflects the inflammatory activity of neutrophils and the regulatory functions of lymphocytes, making it valuable for monitoring acute inflammation and infectious diseases. In summary, the MLR is particularly well-suited for assessing chronic inflammation and immune balance. At the same time, the SII excels in providing a broader perspective on systemic inflammation by incorporating platelet dynamics, and the NLR is highly applicable to the evaluation of acute inflammation and infection-related conditions. Although this study focused primarily on prediction rather than causality, future research should explore the causal association between inflammatory markers and psoriasis using trial emulation approaches. Given that several biomarkers are modifiable, target trial emulation (TTE) frameworks—such as those proposed by Yang et al. (45)—could be used to simulate randomized comparisons in observational data. These methods, such as inverse probability weighting, marginal structural models, and targeted maximum likelihood estimation, can improve causal interpretation and support hypothetical intervention strategies for controlling inflammation and preventing disease.

This study has several limitations that should be noted. First, the cross-sectional design of the NHANES database prevents the dynamic evaluation of variable changes over time, thereby limiting the ability to infer causal relationships between identified risk factors and psoriasis incidence. Furthermore, because both biomarker measurements and psoriasis status were assessed at the same time point in the NHANES dataset, the temporal relationship between predictors and outcome could not be determined. This lack of a defined time window prevents the assessment of biomarker trajectories or lead-time effects. Future studies should employ longitudinal data with clearly defined follow-up windows to explore temporal patterns and establish causal directionality between inflammation and psoriasis onset. Additionally, although adjustments were made for known confounding factors, the possibility of unmeasured or residual confounding cannot be ruled out, which may impact the accuracy of the findings. To address this, future research should consider employing large-scale, multicenter prospective cohort studies to better establish causal links. On the other hand, the diagnosis of psoriasis in this study relied primarily on self-report questionnaire data, introducing potential recall bias and the risk of disease misclassification. Furthermore, there was a significant difference between the psoriasis and non-psoriasis groups. Although the SMOTEEN technique was applied to mitigate this imbalance, the use of synthetic samples may have influenced model performance and generalizability, necessitating further validation. Moreover, as the model was constructed using only NHANES data and has not been externally validated, its generalizability to other populations remains uncertain. Future research should focus on validating the model in independent, regionally diverse cohorts with clinically confirmed outcomes.

## Conclusions

This study utilized machine learning techniques to develop predictive models for psoriasis, with the gradient boosting model demonstrating the best performance among all the tested algorithms. Not only does it achieve the highest predictive accuracy, but it also excels in classification efficacy and feature importance analysis, highlighting key inflammatory markers such as the MLR, SII, and NLR. These markers are particularly important as reliable indicators for systemic inflammation evaluation and psoriasis prediction, emphasizing their potential clinical application. This research provides a solid theoretical foundation and methodological support for psoriasis prediction and personalized disease management.

## Data Availability

The original contributions presented in the study are included in the article/**Supplementary Material**. Further inquiries can be directed to the corresponding author.
