# Supplementary material for: Exploring immune-inflammation markers in psoriasis prediction using advanced machine learning algorithms
Source: Front Immunol. 2025 Jul 31;16:1619490. doi: 10.3389/fimmu.2025.1619490 (PMC12350470; doi:10.3389/fimmu.2025.1619490)
Supplement: Supplementary file 1 [file DataSheet1.pdf]

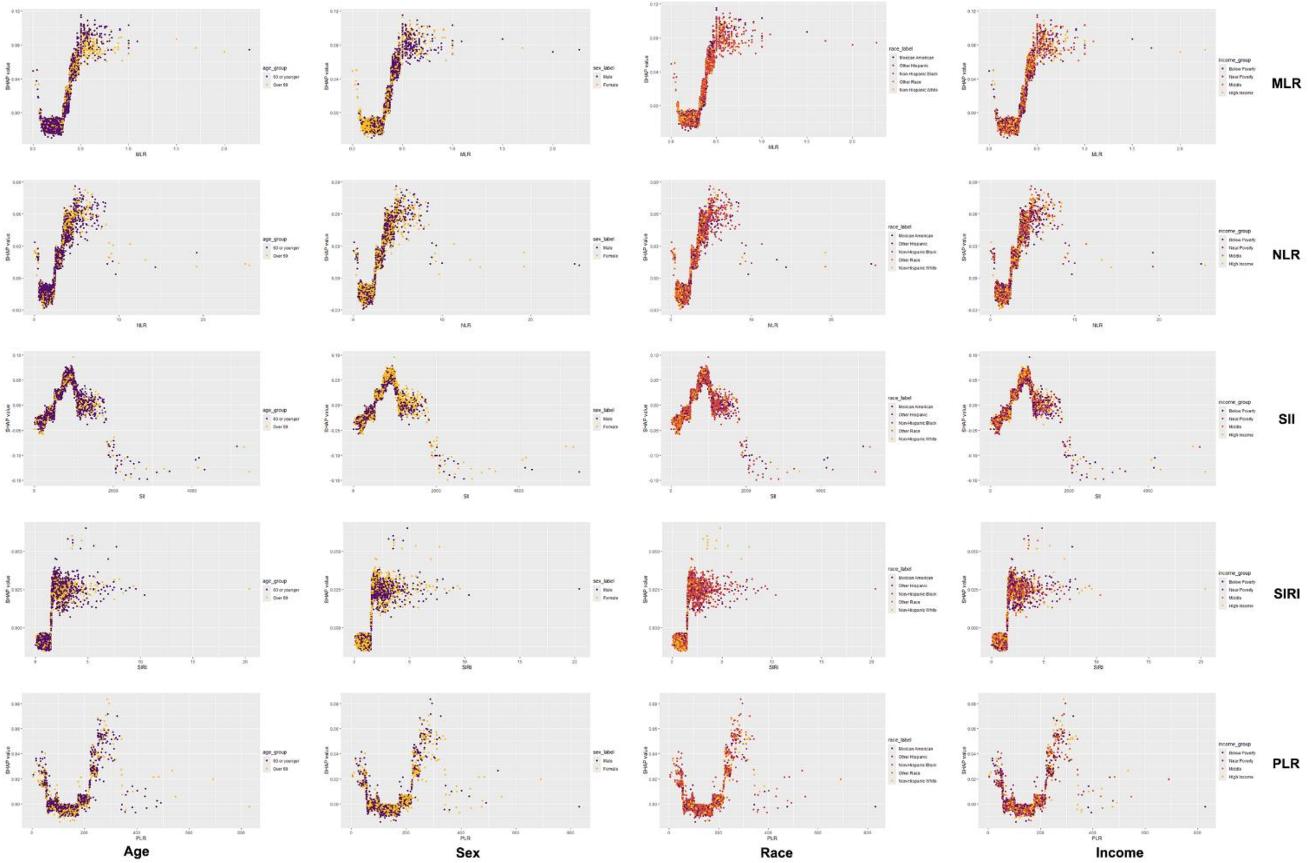


**Figure S1**. SHAP Dependence Plots Stratified by Demographic Factors. SHAP dependence plots display the marginal impact of inflammatory markers including NLR, MLR, SII, SIRI, and PLR on GBM model predictions, with feature contributions stratified by age (≤60 vs. >60 years), sex (Male vs. Female), race/ethnicity (Mexican American, Other Hispanic, Non-Hispanic Black, Other Race, Non-Hispanic White), and income level (Below Poverty [PIR ≤ 1], Near Poverty [1 < PIR ≤ 2], Middle Income [2 < PIR ≤ 4], High Income [PIR > 4]).

Table S1: Proportion of Missing Values for Variables (%).

| | | | | | | | | |
|---|---|---|---|---|---|---|---|---|
| **Demographics** | | | | | | | | |
| SEQN | Age | Race | Educational status | Educational status | Marital Status | Family PIR | | |
| 0 | 0 | 0 | 0 | 3.82 | 3.79 | 7.41 | | |
| **Lifestyle Factors and Comorbidities** | | | | | | | | |
| Drinking habit | Smoking history | hypertension | Diabetes | CVD | OA | | | |
| 10.34 | 2.45 | 2.94 | 45.22 | 29.48 | 29.48 | | | |
| **Health Metrics** | | | | | | | | |
| SBP | DBP | BMI | FPG | HbA1c, % | TC | HDL-C | TG | LDL-C |
| 4.11 | 4.11 | 1.20 | 51.93 | 0.30 | 1.39 | 1.39 | 52.36 | 53.29 |
| Platelet count (1000 cells/uL) | Segmented neutrophils num (1000 cell/uL) | Lymphocyte number (1000 cells/uL) | Monocyte number (1000 cells/uL) | White blood cell count (1000 cells/uL) | | | | |
| 0 | 0 | 0 | 0 | 0 | | | | |

Abbreviations: SEQN: Sequence Number; PIR: Poverty Income Ratio; CVD: Cardiovascular Disease; OA: Osteoarthritis; SBP: Systolic Blood Pressure; DBP: Diastolic Blood Pressure; BMI: Body Mass Index; FPG: Fasting Plasma Glucose; TC: Total Cholesterol; HDL-C: High-Density Lipoprotein Cholesterol; TG: Triglycerides; LDL-C: Low-Density Lipoprotein Cholesterol.

Table S2: Variance Inflation Factors (VIFs) of Inflammatory Markers Indicating Multicollinearity Assessment.

| | | | | |
|---|---|---|---|---|
| Platelet count (1000 cells/uL) | Segmented neutrophils num (1000 cell/uL) | Lymphocyte number (1000 cells/uL) | Monocyte number (1000 cells/uL) | White blood cell count (1000 cells/uL) |
| 2.355189 | 1.621813 | 1.505572 | 1.533146 | 2.333552 |
| NLR | dNLR | SII | SIRI | MLR |
| 2.343043 | 2.020183 | 2.692141 | 2.209523 | 1.727953 |
| PLR | NLMR | AISI | PWR | NPR |
| 1.873981 | 2.367837 | 2.526775 | 1.784441 | 1.387915 |

Abbreviations: NLR: Neutrophil-to-Lymphocyte Ratio; dNLR: Derived Neutrophil-to-Lymphocyte Ratio; SII: Systemic Immune-Inflammation Index; SIRI: Systemic Inflammation Response Index; MLR: Monocyte-to-Lymphocyte Ratio ; PLR: Platelet-to-Lymphocyte Ratio; NLMR: Neutrophil-to-Lymphocyte and Monocyte-to-Lymphocyte Ratio; AISI: Aggregate Index of Systemic Inflammation; PWR: Platelet-to-White Blood Cell Ratio; NPR: Neutrophil-to-Platelet Ratio.

Table S3: Performance Metrics of Machine Learning Models for Classification Analysis

| Model | Accuracy | Precision | Recall | F1_Score | AUC |
|---|---|---|---|---|---|
| Random Forest | 0.773 | 0.977 | 0.785 | 0.871 | 0.617 |
| Neural Network | 0.972 | 0.972 | 1 | 0.986 | 0.5 |

| | | | | | |
|---|---|---|---|---|---|
| XGBoost | 0.792 | 0.976 | 0.806 | 0.883 | 0.615 |
| K-Nearest Neighbors | 0.534 | 0.972 | 0.536 | 0.691 | 0.512 |
| Gradient Boosting | 0.748 | 0.978 | 0.758 | 0.854 | 0.629 |
| Logistic Regression | 0.67 | 0.978 | 0.676 | 0.799 | 0.627 |
| SVM | 0.741 | 0.978 | 0.751 | 0.849 | 0.618 |
| AdaBoost | 0.772 | 0.977 | 0.784 | 0.87 | 0.601 |
| Naive Bayes | 0.718 | 0.978 | 0.726 | 0.833 | 0.608 |

Table S4. Hosmer-Lemeshow Chi-Square Test Results for Model Statistical Significance and Fit Assessment.

| Model | Test Statistic | *P*-Value |
|---|---|---|
| Random Forest | 5.145 | 0.02 |
| Neural Network | 7.933 | < 0.01 |
| XGBoost | 91.689 | < 0.01 |
| K-Nearest Neighbors | 270.057 | < 0.01 |
| Gradient Boosting | 3.023 | 0.08 |

| | | |
|---|---|---|
| Logistic Regression | 1.091 | 0.3 |
| SVM | 12.242 | < 0.01 |
| AdaBoost | 4.227 | 0.04 |
| Naive Bayes | 49.437 | < 0.01 |
